# The complete chloroplast genome of *Hedera helix* (Araliaceae): comparative plastome analyses reveal divergence hotspots, selective constraints, and phylogenetic placement

**DOI:** 10.3389/fgene.2026.1855347

**Published:** 2026-08-06

**Authors:** Dujuan Zhan, Yaqi Fang, Hao Li, Chencheng Zheng, Jin Jiang, Jie Chen

**Affiliations:** 1 Industrial College of Traditional Chinese Medicine and Health, Lishui University, Lishui, China; 2 College of Agriculture and Biotechnology, Lishui University, Lishui, China

**Keywords:** Araliaceae, chloroplast genome, Hedera helix, phylogenomics, ycf1

## Abstract

**Background:**

Hedera helix: is an ecologically, horticulturally, and medicinally important member of Araliaceae. Although a genome sequence of this species has been reported, detailed chloroplast genome annotation and focused comparative plastome analysis of *H. helix* remain limited. This study aimed to assemble and annotate the complete chloroplast genome of *H. helix* and to evaluate its structural features, sequence variation, and phylogenetic position in a comparative family-level context.

**Methods:**

Approximately 22 Gb of Illumina 150 bp paired-end data were generated for *H. helix*. The complete chloroplast genome was assembled *de novo* and annotated using standard plastome assembly and annotation pipelines. Comparative analyses included genome structure characterization, repeat and simple sequence repeat (SSR) detection, codon usage analysis, IR/SC boundary comparison, nucleotide diversity scanning, synteny assessment, and plastome-based phylogenetic reconstruction using representative Araliaceae taxa.

**Results:**

The *H. helix* plastome is 156,688 bp in length and exhibits the typical quadripartite structure, comprising a large single-copy region of 86,626 bp, a small single-copy region of 18,180 bp, and two inverted repeats of 25,941 bp each, with an overall GC content of 37.99%. A total of 132 genes were annotated, including 87 protein-coding genes, 37 tRNAs, and 8 rRNAs. Repeat analyses identified 54 long repeats and 47 SSRs, most of which were A/T-rich. Codon usage analysis revealed a preference for codons ending in A or T. Comparative analyses showed that plastome structure and gene order were highly conserved across sampled Araliaceae species, with only minor variation at IR/SC junctions. Most protein-coding genes had Ka/Ks ratios consistent with purifying selection, whereas nucleotide diversity analysis identified several localized divergence hotspots, particularly in *ycf1*. Phylogenomic analysis based on plastome data strongly supported *H. helix* as sister to (*H. rhombea* + *H. nepalensis* var. *sinensis*).

**Conclusion:**

This study provides a complete and curated plastome resource for *H. helix* and shows that its chloroplast genome is structurally conserved relative to other sampled Araliaceae plastomes. The identified SSRs and highly variable regions, especially *ycf1*, represent potential candidates for future marker development. In addition, the plastome-based phylogenetic results provide useful evidence for evaluating the placement of *Hedera* within Araliaceae.

## 1 Introduction

Araliaceae is a diverse angiosperm family with high ecological and economic value, including many woody species and several medicinally important plants. In recent years, plastome data have become an important genomic resource for this family, improving phylogenetic resolution across major lineages and providing a shared framework for comparative genomics and marker development (Kang et al., 2023; Yan et al., 2023). *Hedera helix* (common ivy) is one of the best-known Araliaceae species and is widely cultivated as an ornamental climber, while its leaves are also used in herbal medicine; the European Medicines Agency recognizes ivy leaf preparations as an expectorant for productive cough (European Medicines Agency, 2017). These horticultural and medicinal uses increase the need for reliable identification tools and stable genomic references.

Chloroplast genomes are widely used in plant systematics and evolution. They have a relatively conserved structure and gene content in most angiosperms. A typical plastome is a circular DNA molecule with a quadripartite structure. It contains a large single-copy (LSC) region, a small single-copy (SSC) region, and two inverted repeats (IRs) (Daniell et al., 2016). This conserved architecture supports robust genome assembly and enables direct comparisons among related taxa (Daniell et al., 2016). Although plastomes contain informative variation, their overall evolutionary rate is often lower than that of many nuclear regions, which can limit resolution when only a few loci are used. Standard barcode loci may not separate closely related species in some lineages (Dong et al., 2012). Whole plastomes provide a larger number of characters and a richer source of variation (Daniell et al., 2016). They also allow researchers to scan the genome and find regions with high nucleotide diversity (Dong et al., 2012). These regions are useful for low-level phylogeny, population studies, and authentication (Dong et al., 2012).

Several plastid loci have been evaluated for plant barcoding and phylogenetics. Genome-wide scans have shown that variability is uneven across the plastome (Dong et al., 2012). Some coding and intergenic regions evolve faster than others, among which *ycf1* is a well-supported example because large-scale tests have shown that it contains highly variable segments with good performance for species discrimination in land plants (Dong et al., 2015). This makes *ycf1* a strong candidate for targeted marker development when common loci show weak resolution (Dong et al., 2012; Dong et al., 2015). Plastomes also support the discovery of simple sequence repeats (SSRs). Although chloroplast SSRs can be useful markers for population genetics and for tracing maternal lineages, their interpretation may be complicated by homoplasy and lineage-specific mutation patterns (Wheeler et al., 2014). A plastome-based workflow can still provide a practical way to catalogue SSRs and other repeats. It can also help prioritize a small set of robust loci for follow-up testing (Wheeler et al., 2014; Yan et al., 2023).

In Araliaceae, plastome sampling has grown quickly, but coverage is still uneven among genera and species. Recent work has used broad plastome sampling to address difficult nodes and rapid radiations (Kang et al., 2023). Comparative plastome studies in Araliaceae also emphasize marker screening and whole-plastome phylogenomic analyses (Yan et al., 2023). However, plastome data alone can reflect only the maternal history in many angiosperms. This can lead to discordance with nuclear signals in cases of rapid diversification or hybridization (Kang et al., 2023). For this reason, curated plastome resources remain valuable, but they should be interpreted within a careful evolutionary context (Kang et al., 2023).

Although a genome sequence of *H*. *helix* has been reported previously (Christenhusz et al., 2023), detailed plastome annotation and focused comparative analysis remain useful for marker development and cross-study comparison. In this study, we provide a curated chloroplast genome of *H. helix* from a Chinese sample and place it within a comparative plastome framework for Araliaceae. Rather than simply reporting another plastid sequence, we focus on genome organization, repeat and SSR features, IR/SC boundary variation, nucleotide diversity hotspots, selective constraints, and plastome-based phylogenetic placement. These analyses provide additional information for marker development, species identification, and comparative plastome studies in *Hedera* and related Araliaceae taxa.

## 2 Materials and methods

### 2.1 Sampling and sequencing

Leaves of *H*. *helix* were sampled from a locality in the vicinity of Anshun, Guizhou Province, China (26°14′56″N, 105°56′06″E). The plant material was formally identified by Dujuan Zhan, and a voucher specimen (LSU_20250519) was prepared and deposited in the Herbarium of Lishui University. Total genomic DNA was isolated from fresh leaf material using the Rapid Plant Genomic DNA Isolation Kit (Sangon Biotech, Shanghai, China) according to the manufacturer’s protocol. The extracted DNA was subsequently used to construct Illumina libraries and sequenced on an Illumina HiSeq 2,500 platform with 150 bp paired-end reads, yielding short-read data for downstream analyses.

### 2.2 Plastome assembly and annotation

Approximately 22 Gb of 150 bp paired-end Illumina reads were produced for plastome reconstruction. Raw reads were quality-filtered by removing adapter sequences and trimming low-quality bases using Trimmomatic v0.39 (Bolger et al., 2014). The plastome was *de novo* assembled with GetOrganelle v1.8.0.1 (Jin et al., 2020) using the embryophyte plastid mode, with the main parameters -F embplant_pt -R 15 -k 21,45,65,85,105 -t 12. The word size was automatically estimated by GetOrganelle, and other relevant settings, including minimum read overlap, were kept at their default values. The circular plastome sequence generated from the selected K105 assembly graph was adopted as the final complete chloroplast genome. To assess assembly quality, clean reads were mapped back to the assembled chloroplast genome. Per-site sequencing depth was calculated using SAMtools depth, and genome coverage, minimum and maximum coverage, and read-mapping statistics were summarized using SAMtools (Li et al., 2009). The plastome-wide depth distribution was visualized in Supplementary Figure S1. The final assembly graph was visualized using Bandage (Wick et al., 2015) to assess the circular assembly structure (Supplementary Figure S2), and the IR/SC junctions were further checked by read-depth analysis and manual inspection of the annotated boundary regions (Supplementary Figure S3). Genome annotation was conducted using GeSeq (Tillich et al., 2017) and CPGAVAS2 (Shi et al., 2019) to identify protein-coding genes (PCGs), rRNAs, and tRNAs and to determine IR boundaries and genome orientation. The annotation was subsequently inspected and manually curated in CPGView (Liu et al., 2023), with particular attention to exon-intron structures and the identification and visualization of cis- and trans-spliced genes.

### 2.3 Plastome characteristics

The overall nucleotide composition (GC content) of the finalized plastome was calculated in EditSeq v7.1.0 (Burland, 2000). A graphical circular map of the *H*. *helix* plastome was drawn with OGDRAW v1.3.1 (Lohse et al., 2007) based on the curated annotation, showing the LSC, SSC, and IR regions and the distribution of annotated gene features.

### 2.4 Repeat and simple sequence repeat (SSR) analyses

Interspersed repeat sequences in the *H*. *helix* plastome were surveyed using REPuter (Kurtz et al., 2001), including forward, reverse, complement, and palindromic repeats. The search was performed with a minimum repeat length of 30 bp, a maximum repeat length of 100 bp, and a Hamming distance of 3. Microsatellites (SSRs) were identified with MISA (Beier et al., 2017) using minimum repeat thresholds of 10, 5, 4, 3, 3, and 3 for mono-, di-, tri-, tetra-, penta-, and hexanucleotide motifs, respectively; repeats separated by ≤100 bp were regarded as compound SSRs.

### 2.5 Analysis of codon preference

Codon usage patterns in *H. helix* were evaluated by calculating codon frequencies and relative synonymous codon usage (RSCU) values using CodonW v1.4.4 (Sharp and Li, 1987). All annotated protein-coding sequences were included in the analysis under the bacterial, archaeal, and plant plastid genetic code, corresponding to translation table 11, and termination codons were excluded. The resulting RSCU dataset was subsequently plotted to facilitate visualization and interpretation of codon preference.

### 2.6 Analysis of IR boundary

The boundaries between the LSC, SSC, and IR regions (JLB, JSB, JSA, and JLA) were examined using IRscope v3.1 (Amiryousefi et al., 2018) to evaluate IR expansion/contraction patterns. The curated plastomes of *H*. *helix* (PX328984.1) and five related Araliaceae species, including *Hedera nepalensis var. sinensis* (MK130890.1), *Dendropanax dentigerus* (KP271241.1), *Eleutherococcus brachypus* (MN527993.1), *Metapanax delavayi* (KC456165.1), and *Kalopanax septemlobus* (KC456167.1), were imported into IRscope, and the positions of the four junctions and their flanking genes were compared among taxa.

### 2.7 Analysis of Ka/Ks ratios

Orthologous PCGs were identified using the *H. helix* plastome as the reference and five related Araliaceae plastomes as queries. Coding sequences and their translated protein sequences were extracted in PhyloSuite v1.2.3 (Zhang et al., 2020). Candidate homologs were identified by BLASTP searches using BLAST + v2.10.1 (Camacho et al., 2009) against the *H. helix* proteome, and the top-scoring match was retained for each gene. Protein sequences were aligned with MAFFT v7.526 (Katoh and Standley, 2013), and the corresponding codon alignments were generated with PAL2NAL v14 (Suyama et al., 2006). Pairwise nonsynonymous (Ka) and synonymous (Ks) substitution rates were then estimated with KaKs_Calculator 3.0 (Zhang, 2022) under the YN model (Yang and Nielsen, 2000), and the resulting Ka/Ks ratios were used to assess selective constraints among shared plastid PCGs.

### 2.8 Whole-plastome sequence similarity analysis

Whole-plastome sequence similarity was assessed with mVISTA in Shuffle-LAGAN mode (Frazer et al., 2004), using the *H*. *helix* plastome as the reference and five related Araliaceae plastomes as comparators.

### 2.9 Structural collinearity analysis

To assess large-scale structural variation among *H. helix* and related Araliaceae plastomes, the complete chloroplast genomes of *H. helix*, *H. nepalensis* var. *sinensis*, *Dendropanax dentigerus*, *Eleutherococcus brachypus*, *Metapanax delavayi*, and *Kalopanax septemlobus* were compared using progressiveMauve (Darling et al., 2010). Before alignment, all plastomes were normalized to the *psbA* starting position to minimize differences caused by alternative circular-genome starting points. Locally collinear blocks were then examined to detect potential large-scale rearrangements or inversions.

### 2.10 Nucleotide diversity analysis

Nucleotide diversity (Pi) was estimated in DnaSP v6 (Rozas et al., 2017) based on a sliding-window analysis, with a window length of 600 bp and a step size of 200 bp. Windows with Pi values > 0.01 were treated as candidate hypervariable regions. This empirical cutoff was chosen based on the Pi distribution in the present dataset, because it was approximately twice the genome-wide mean Pi value and allowed the identification of localized regions with clearly elevated nucleotide diversity. The coordinates, Pi values, and associated genes or intergenic regions of these windows are provided in Supplementary Table S1.

### 2.11 Phylogenetic relationship analysis

The final phylogenetic dataset comprised 28 representative Araliaceae plastomes plus *Torricellia tiliifolia* as the outgroup (29 taxa in total). Sampling was designed to represent the major plastome lineages relevant to the placement of *H. helix* rather than to provide an exhaustive census of public Araliaceae plastome accessions. Detailed taxon information and GenBank accession numbers are provided in Supplementary Table S2. All plastome sequences were imported into PhyloSuite v1.2.3 (Zhang et al., 2020) and standardized to a consistent orientation and start position prior to downstream analyses. Multiple sequence alignment was performed using MAFFT v7.526 (Katoh and Standley, 2013) under the auto strategy. The aligned matrix was then used to reconstruct a maximum-likelihood (ML) phylogeny using IQ-TREE v2 (Minh et al., 2020). The best-fit nucleotide substitution model was selected using ModelFinder (Kalyaanamoorthy et al., 2017) according to the Bayesian information criterion (BIC), and K3Pu + F + I + R5 was chosen. Branch support values shown on the tree were assessed using the SH-aLRT test with 1,000 replicates in IQ-TREE, and nodes with SH-aLRT values ≥80% were interpreted as well supported. The resulting topology and SH-aLRT support values were formatted and annotated in R using ggtree (Xu et al., 2022).

## 3 Results

### 3.1 Plastome organization and gene content

**FIGURE 1:**
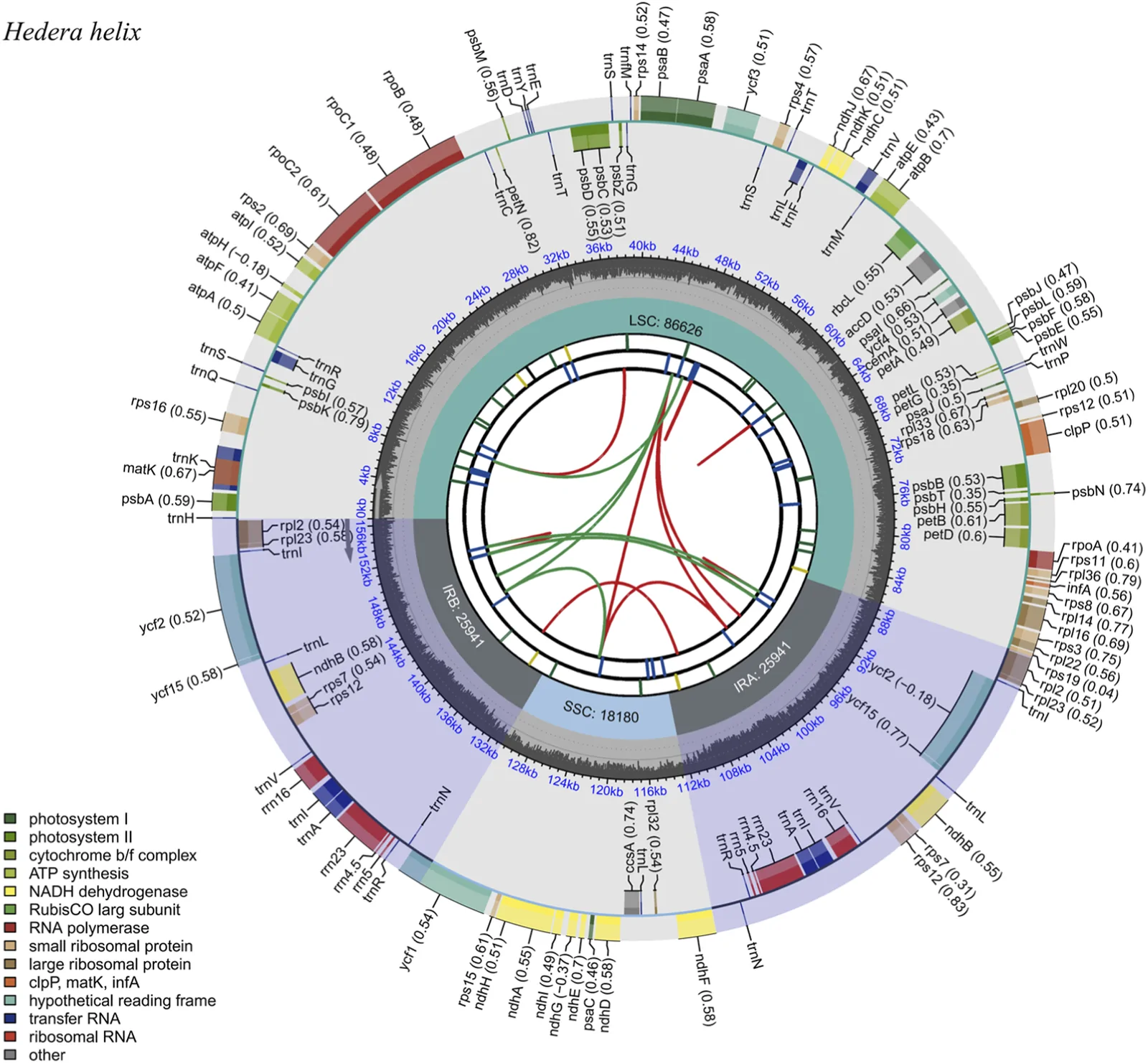
Plastome map of *Hedera helix*. The inner circle denotes GC content (dark grey) and AT content (light grey). Genes on the outer circle are colour-coded according to functional categories.

The complete chloroplast genome of *H. helix* is 156,688 bp in length and exhibits the typical quadripartite structure, consisting of a large single-copy (LSC) region (86,626 bp), a small single-copy (SSC) region (18,180 bp), and a pair of inverted repeats (IRa/IRb; 25,941 bp each). The overall GC content is 37.99%, with higher GC content in the IR regions (43.07%) than in the LSC (36.19%) and SSC (32.05%) regions (Figure 1). Read-mapping analysis showed 100.0% genome coverage and continuous read depth across the plastome, with an average depth of 9,860.09× and a depth range of 2,227× to 19904× (Supplementary Figure S1). A total of 10,463,888 mapped alignments were obtained, corresponding to a mapping rate of 6.55%. Bandage visualization of the selected GetOrganelle assembly graph supported a circular plastome structure (Supplementary Figure S2), and the four IR/SC junctions were also supported by continuous read coverage (Supplementary Figure S3).

A total of 132 genes were annotated in the plastome (114 unique genes), including 87 protein-coding genes (80 unique), 37 tRNA genes (30 unique), and 8 rRNA genes (4 unique) (Table 1). The difference between the total and unique gene numbers is attributable to duplicated copies within the IRs. Intron-containing genes were common: A total of 18 unique genes contained introns, including 15 genes with one intron and three genes with two introns. The three two-intron genes were *clpP*, *rps12*, and *ycf3*; *rps12* was annotated as a trans-spliced gene with separated exons. Most protein-coding genes initiated with the canonical ATG start codon; however, after manual verification, non-ATG start codons were retained in several genes, including *petN* (ATT), *psbC* (GTG), *psbT* (ATC), *rps19* (GTG), *ycf15* (GTG; including the duplicated copy), *ndhD* (ATA), and *ycf1* (GTG).

**TABLE 1:** Gene composition of the plastome of *Hedera helix*.

Category	Gene group	Gene name	Number
Photosynthesis	Subunits of photosystem I	*psaA, psaB, psaC, psaI, psaJ*	5
	Subunits of photosystem II	*psbA, psbB, psbC, psbD, psbE, psbF, psbH, psbI, psbJ, psbK, psbL, psbM, psbN, psbT, psbZ*	15
	Subunits of NADH dehydrogenase	ndhA*, ndhB*, ndhC, ndhD, ndhE, ndhF, ndhG, ndhH, ndhI, ndhJ, ndhK	12
	Subunits of cytochrome b6/f complex	petA, petB*, petD*, petG, petL, petN	6
	Subunits of ATP synthase	atpA, atpB, atpE, atpF*, atpH, atpI	6
	Large subunit of rubisco	*rbcL*	1
Self-replication	Proteins of large ribosomal subunit	rpl14, rpl16*, rpl2*, rpl20, rpl22, rpl23, rpl32, rpl33, rpl36	11
	Proteins of small ribosomal subunit	rps11, rps12**, rps14, rps15, rps16*, rps18, rps19, rps2, rps3, rps4, rps7, rps8	14
	Subunits of RNA polymerase	rpoA, rpoB, rpoC1*, rpoC2	4
	Ribosomal RNAs	*rrn16, rrn23, rrn4.5, rrn5*	8
	Transfer RNAs	trnA-UGC*, trnC-GCA, trnD-GUC, trnE-UUC, trnF-GAA, trnG-GCC, trnG-UCC*, trnH-GUG, trnI-CAU, trnI-GAU*, trnK-UUU*, trnL-CAA, trnL-UAA*, trnL-UAG, trnM-CAU, trnN-GUU, trnP-UGG, trnQ-UUG, trnR-ACG, trnR-UCU, trnS-GCU, trnS-GGA, trnS-UGA, trnT-GGU, trnT-UGU, trnV-GAC, trnV-UAC*, trnW-CCA, trnY-GUA, trnfM-CAU	37
Other genes	Maturase	*matK*	1
	Protease	clpP**	1
	Envelope membrane protein	*cemA*	1
	Acetyl-CoA carboxylase	*accD*	1
	c-type cytochrome synthesis gene	*ccsA*	1
	Translation initiation factor	*infA*	1
Genes of unknown Function	Conserved hypothetical chloroplast ORF	ycf1, ycf15, ycf2, ycf3**, ycf4	7

*Gene with one intron; **gene with two introns.

For comparison, the plastid genome associated with the chromosome-level genome assembly of *H. helix* reported by Christenhusz et al. (2023) is 162,225 bp in length (OX381724; Supplementary Table S3), whereas the circular chloroplast genome assembled in the present study is 156,688 bp (PX328984). To clarify this size difference, we performed a whole-plastome comparison between the two sequences (Supplementary Figure S4). After removal of the redundant 5,537-bp terminal overlap from OX381724 and rotation to the same starting position as PX328984, the one-to-one alignment covered 100.0% of PX328984 and showed 99.87% nucleotide identity. Only 56 SNPs and 25 indel events were detected between the two nonredundant plastome sequences, including 12 insertion events and 13 deletion events, corresponding to 144 indel-associated bases in total. In addition, the terminal 6,056-bp segment of the linear OX381724 assembly aligned with 100% identity to two IR-related regions in PX328984, corresponding to positions 86,627-92,682 bp and 150,633-156,688 bp. These quantitative results indicate that the 2 *H. helix* plastomes are nearly identical across their shared sequence regions, and that the apparent size difference is best explained by a redundant IR-derived terminal segment retained in OX381724 rather than by a true expansion of the LSC or SSC region or by a novel insertion. Read-mapping analyses further supported the circular structure and IR boundary configuration of PX328984, with continuous sequencing depth across the whole plastome and the four IR/SC junctions (Supplementary Figures S1 and S3).

### 3.2 Repeat sequences and SSR analysis

**FIGURE 2:**
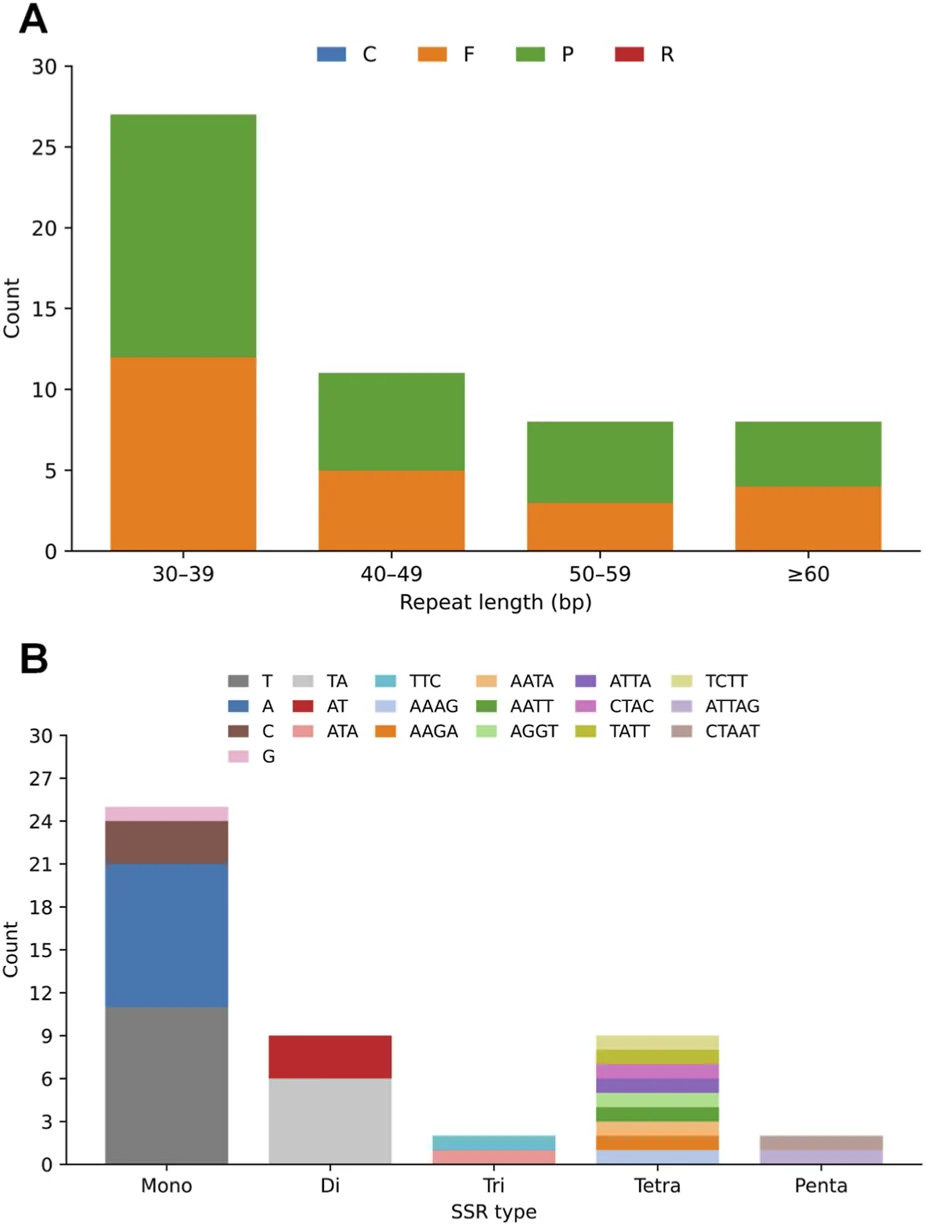
Repeat sequence analyses in the *Hedera helix* plastome. **(A)** Numbers and types of long interspersed repeats. **(B)** Numbers and motif types of simple sequence repeats (SSRs).

Long interspersed repeats (≥30 bp) were identified in the *H. helix* plastome, comprising 54 repeats in total. Palindromic repeats (P, 30) were slightly more abundant than forward repeats (F, 24), whereas reverse (R) and complement (C) repeats were not detected. When binned by repeat length, the 30–39 bp class accounted for the largest proportion (27/54, 50.00%), followed by 40–49 bp (11/54, 20.37%), 50–59 bp (8/54, 14.81%), and ≥60 bp (8/54, 14.81%). The longest repeats reached 79 bp, indicating the presence of moderately long dispersed repeat elements in the chloroplast genome (Figure 2A).

SSRs were also detected and summarized by repeat-unit type (Figure 2B; Supplementary Table S4). A total of 47 SSRs were identified, dominated by mononucleotide repeats (25/47, 53.19%), followed by dinucleotide (9/47, 19.15%) and tetranucleotide repeats (9/47, 19.15%). Trinucleotide and pentanucleotide SSRs were relatively rare (2/47, 4.26% each), and hexanucleotide SSRs were not observed. Among mononucleotide SSRs, A/T-rich motifs predominated (A = 10; T = 11; 21/25, 84.00%), while C (3) and G (1) occurred at much lower frequencies. Dinucleotide SSRs were mainly TA (6) and AT (3), whereas the few trinucleotide SSRs comprised ATA and TTC (one each). Tetranucleotide SSRs were represented by multiple low-frequency motifs (each occurring once), and the pentanucleotide SSRs included ATTAG and CTAAT (one each) (Figure 2B).

### 3.3 Codon usage of the chloroplast genome

**FIGURE 3:**
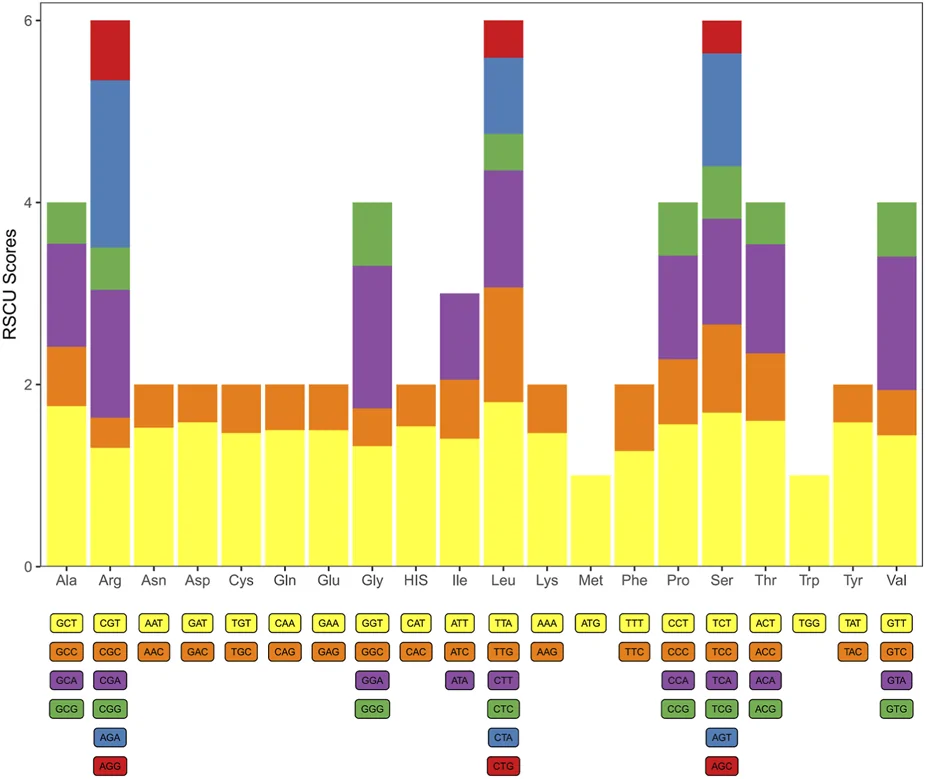
Relative synonymous codon usage (RSCU) of chloroplast protein-coding genes in *Hedera helix*. Codons are grouped by amino acid, and stacked bars represent RSCU values for synonymous codons (codon labels shown below). Values >1 indicate preferentially used codons, whereas values <1 indicate less frequently used codons. The single-codon families Met (AUG) and Trp (UGG) have RSCU values of 1.0.

Codon usage was assessed based on the protein-coding sequences of the *H. helix* plastome (Figure 3). Overall, codons showed a clear preference for A/T-ending codons (A/U-ending in mRNA), as reflected by the third codon position composition (T: 37.78%, A: 31.53%, G: 16.42%, C: 14.28%), with A + T accounting for 69.31% of all third-position bases. Relative synonymous codon usage (RSCU) further supported this AT bias. Twenty-nine codons exhibited RSCU > 1.0, and nearly all of them ended with A or T, indicating preferential usage of A/T-ending synonymous codons. Among synonymous families, several codons showed strong preference, including AGA (Arg; RSCU = 1.838), TTA (Leu; 1.809), GCT (Ala; 1.764), and TCT (Ser; 1.689). In contrast, many GC-ending codons were underrepresented, such as CGC (Arg; RSCU = 0.330), AGC (Ser; 0.359), and CTC (Leu; 0.404). As expected, Met (ATG) and Trp (TGG) (single-codon amino acids) showed no synonymous bias (RSCU = 1.0).

### 3.4 IR boundary comparison

**FIGURE 4:**
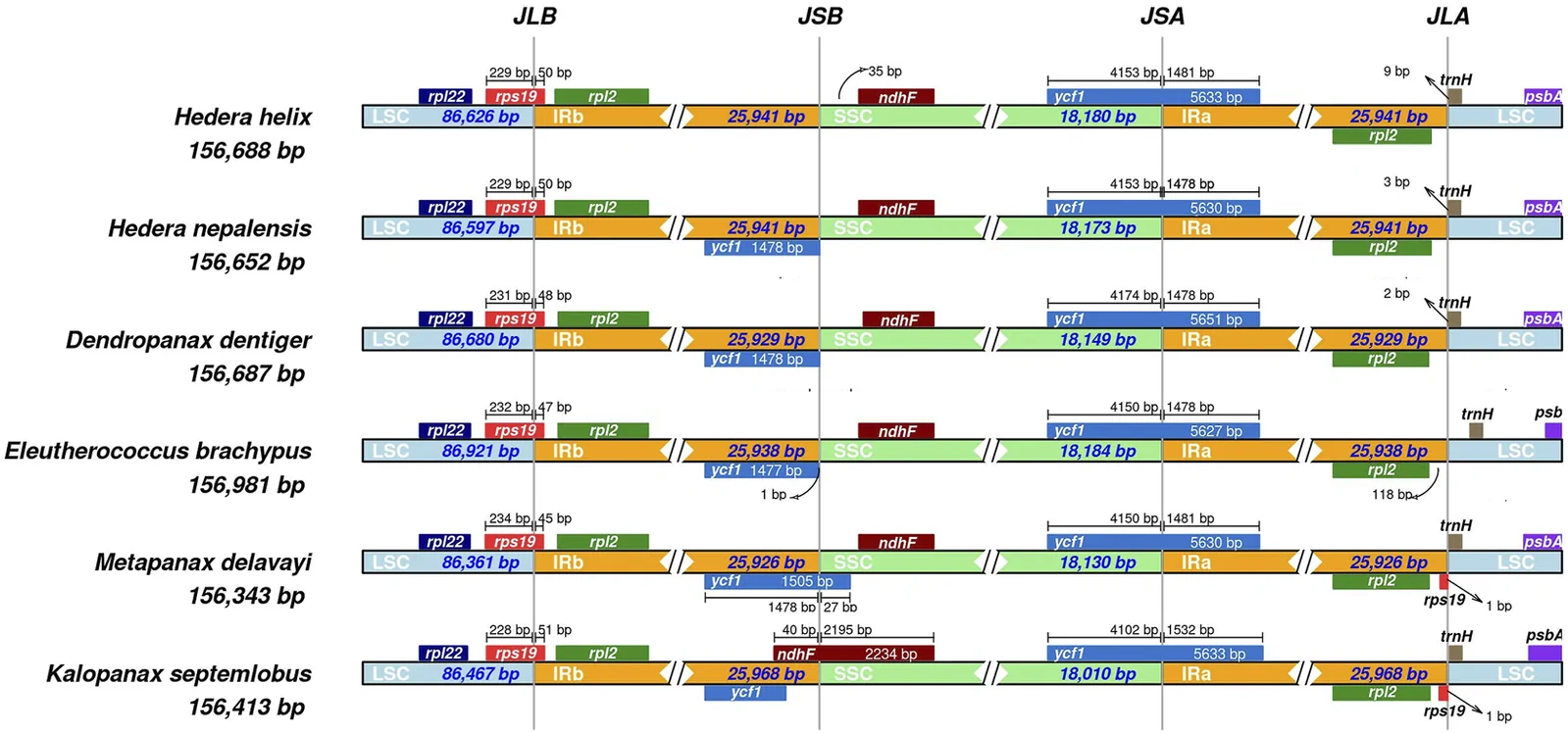
Comparison of LSC, SSC, and IR junctions among six plastomes. The positions of the four boundary regions (JLB, JSB, JSA, and JLA) and their adjacent genes are shown to illustrate IR expansion/contraction across species.

The six Araliaceae plastomes shared a typical quadripartite organization, and the IR boundary regions were overall highly conserved among the sampled taxa (Figure 4). Genome sizes ranged from 156,343 bp (Metapanax delavayi) to 156,981 bp (Eleutherococcus brachypus), with the LSC spanning 86,361–86,921 bp, the SSC 18,010–18,184 bp, and each IR 25,926–25,968 bp, indicating only minor IR expansion/contraction events.

At the JLB (LSC/IRb) junction, rps19 consistently straddled the boundary in all species, with ∼228–234 bp located in the LSC and ∼45–51 bp extending into IRb, while rpl22 remained in the LSC and rpl2 in IRb, reflecting a stable boundary configuration. At the JSB (IRb/SSC) junction, boundary configurations differed subtly among taxa. In *H. helix*, ndhF was located at the SSC proximal end but was separated from the JSB by ∼35 bp, indicating a short intergenic spacer rather than a junction-spanning gene. In contrast, only Kalopanax septemlobus showed ndhF spanning the JSB, with 40 bp extending into IRb and 2,195 bp residing in SSC (total 2,235 bp). A truncated *ycf1* fragment was consistently detected at the IRb end, measuring 1,477–1,478 bp in most taxa; however, in Metapanax delavayi the fragment was longer (1,505 bp) and crossed the JSB (IRb 1,478 bp; SSC 27 bp), whereas Eleutherococcus brachypus exhibited a 1 bp offset at this boundary. The JSA (SSC/IRa) junction was characterized by *ycf1* spanning the boundary in all taxa, with ∼4.10–4.17 kb of the gene located in the SSC and ∼1.48–1.53 kb duplicated within IRa, consistent with the major divergence hotspot detected in *ycf1* in sliding-window analyses. At JLA (IRa/LSC), trnH was consistently located in the LSC and positioned very close to the junction (approximately 2–9 bp), whereas small interspecific differences were observed in the terminal spacing of IRa genes (e.g., increased separation of rpl2 from JLA in E. brachypus). Collectively, these results indicate that IR boundaries are largely conserved across the six plastomes, with only minor bp-level boundary shifts contributing to modest variation in region lengths.

### 3.5 Ka/Ks analysis of plastid protein-coding genes

**FIGURE 5:**
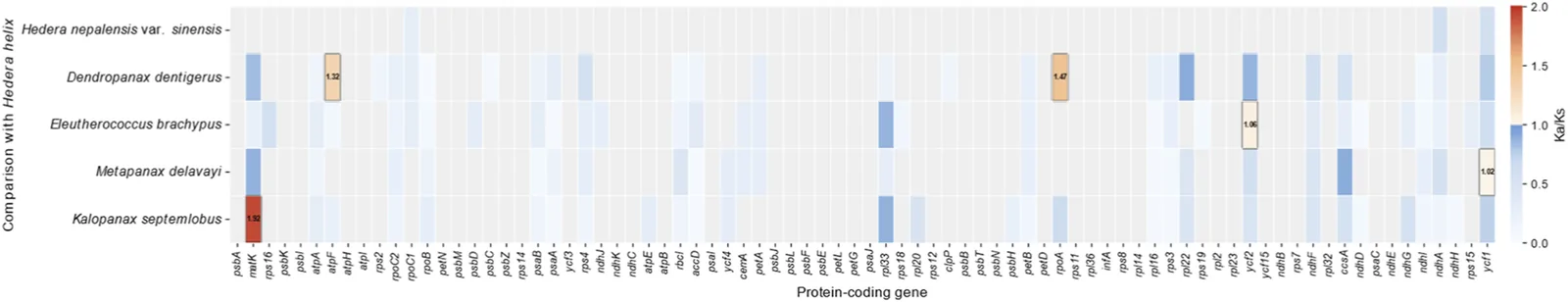
Heatmap of pairwise Ka/Ks ratios for shared chloroplast protein-coding genes among six Araliaceae plastomes. Columns represent shared genes, and rows represent pairwise comparisons between *Hedera helix* and five related taxa (*Hedera nepalensis* var. *Sinensis*, *Dendropanax dentigerus*, *Eleutherococcus brachypus*, *Metapanax delavayi*, and *Kalopanax septemlobus*). Warmer colors indicate higher Ka/Ks values. Gray cells denote undefined estimates after KaKs_Calculator 3.0 filtering, mainly because reliable ratios could not be calculated for comparisons with Ks = 0, identical or nearly identical coding sequences, or insufficient informative substitutions. Outlined cells with labels indicate Ka/Ks > 1.

Pairwise Ka/Ks analysis of shared chloroplast protein-coding genes across the six Araliaceae plastomes showed that most genes had Ka/Ks values below 1, consistent with pervasive purifying selection in plastid PCGs (Figure 5). Only five gene-comparison combinations exceeded 1.0: *matK* in *H. helix* vs. *Kalopanax septemlobus* (Ka/Ks = 1.92), *rpoA* and *atpF* in *H. helix* vs. *Dendropanax dentigerus* (1.47 and 1.32, respectively), *ycf2* in *H. helix* vs. *Eleutherococcus brachypus* (1.06), and *ycf1* in *H. helix* vs. *Metapanax delavayi* (1.02). No gene showed consistently elevated Ka/Ks values across all comparisons. These elevated ratios were restricted to isolated pairwise contrasts and were therefore treated as candidate loci with unusual substitution patterns rather than as evidence of statistically supported positive selection.

### 3.6 Comparative analysis of plastomes

**FIGURE 6:**
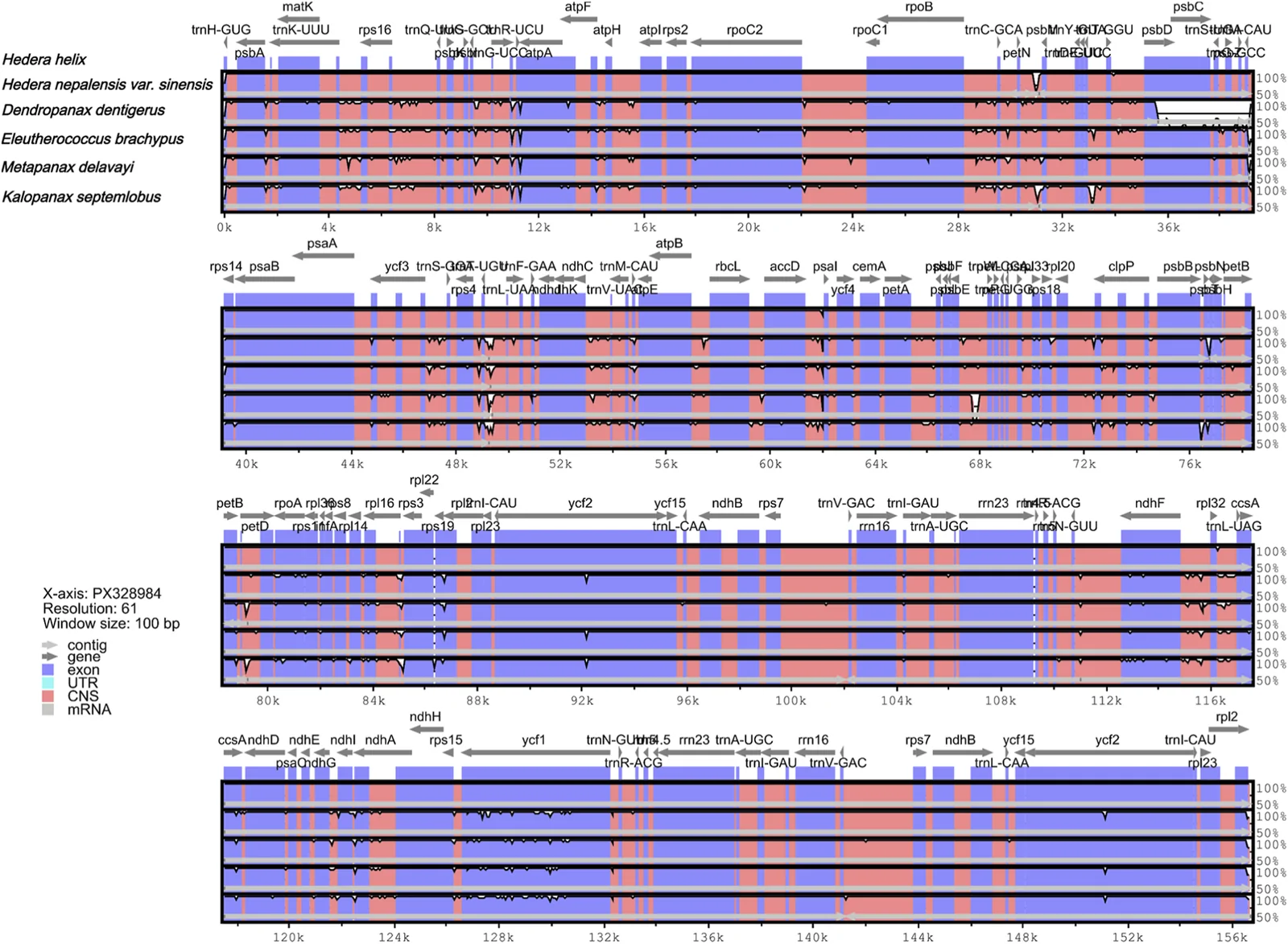
Whole-plastome sequence comparison of six plastomes generated in mVISTA (Shuffle-LAGAN mode). The grey arrows indicate gene orientation, and the thick black bars above the alignment denote the positions of the IR regions. The y-axis shows percentage identity (50%–100%). Genomic features are colour-coded as protein-coding exons (purple), ribosomal RNAs (cyan), and conserved non-coding sequences (CNS; pink).

The overall sequence conservation among the six Araliaceae plastomes was visualized using mVISTA, with *H. helix* as the reference (Figure 6). The plastomes showed a high level of similarity across most regions, with identity values generally remaining close to the upper range (approximately 90%–100%). Coding regions were more conserved than non-coding regions, and most pronounced reductions in sequence identity were concentrated in intergenic spacers and intron-containing loci. In contrast, the IR-associated regions, particularly the rRNA gene cluster, exhibited markedly higher conservation with minimal divergence across all comparisons. Several localized divergence regions were apparent in the LSC and SSC portions of the plastome, including segments surrounding *ycf1* and parts of the *ndh* region, which showed repeated reductions in identity across multiple taxa. Overall, the mVISTA profiles indicate that sequence variation among the sampled Araliaceae plastomes is mainly enriched in non-coding regions and a limited number of comparatively variable loci.

Structural collinearity analysis using progressiveMauve showed that the six compared Araliaceae plastomes were largely collinear after normalization to the *psbA* starting position. No major rearrangements or inversions were detected among *H. helix*, *H. nepalensis* var. *sinensis*, *Dendropanax dentigerus*, *Eleutherococcus brachypus*, *Metapanax delavayi*, and *Kalopanax septemlobus* (Supplementary Figure S5). This result indicates that the plastome organization of *H. helix* is structurally conserved relative to the selected related Araliaceae plastomes. A supplementary comparison of the three available *Hedera* plastomes (*H. helix*, *H. rhombea*, and *H. nepalensis* var. *sinensis*) showed highly similar genome sizes (156,652-156,688 bp), GC contents (37.99%–38.00%), IR lengths (25,941 bp), and gene content (132 genes), with only minor differences in SSR abundance and localized variable regions (Supplementary Table S5).

**FIGURE 7:**
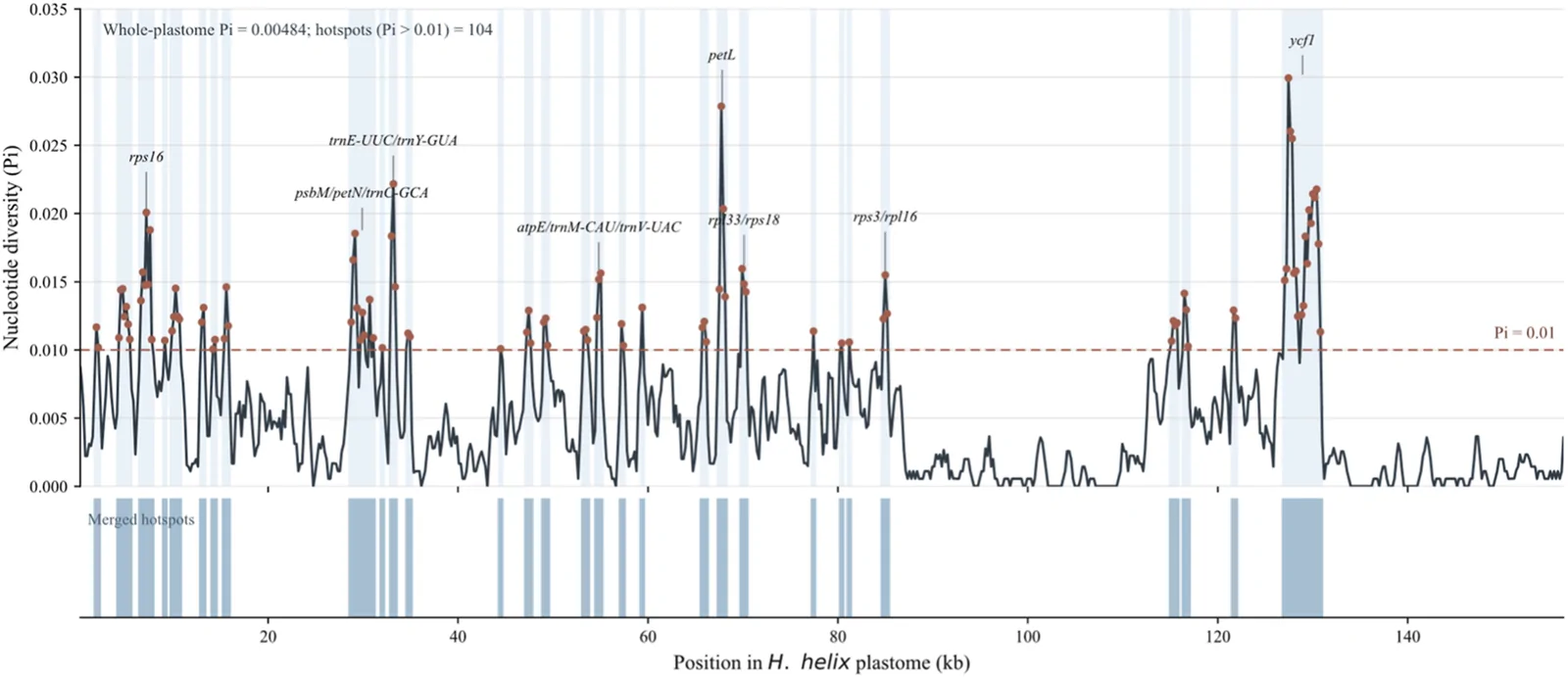
Sliding-window analysis of nucleotide diversity (Pi) across six Araliaceae plastomes. Pi was calculated with a window length of 600 bp and a step size of 200 bp. Coordinates are based on the *Hedera helix* plastome. The dashed line indicates the hotspot threshold (Pi = 0.01).

Nucleotide diversity (Pi) across the six Araliaceae plastomes was assessed using a sliding-window analysis, with a window length of 600 bp and a step size of 200 bp (Figure 7). Overall sequence divergence was low, with a genome-wide mean Pi of 0.00484, indicating a largely conserved plastome background. However, sequence variation was unevenly distributed along the plastome, and 104 windows exceeded the empirical hotspot threshold of Pi > 0.01 (Supplementary Table S1). The strongest hotspot was located within *ycf1*, spanning 126,745–131,105 bp, with a maximum window Pi of 0.02994. A second major hotspot occurred in the *petL* region (67,215–68,410 bp; maximum Pi = 0.02786). Additional variable regions were detected in or near *rps16*, *trnE-UUC/trnY-GUA*, *psbM/petN/trnC-GCA*, *trnT-UGU*, *rpl33/rps18*, and *rps3/rpl16*. Together, these regions represent candidate loci for future comparative and phylogenetic studies in Araliaceae.

### 3.7 Phylogenetic analysis

**FIGURE 8:**
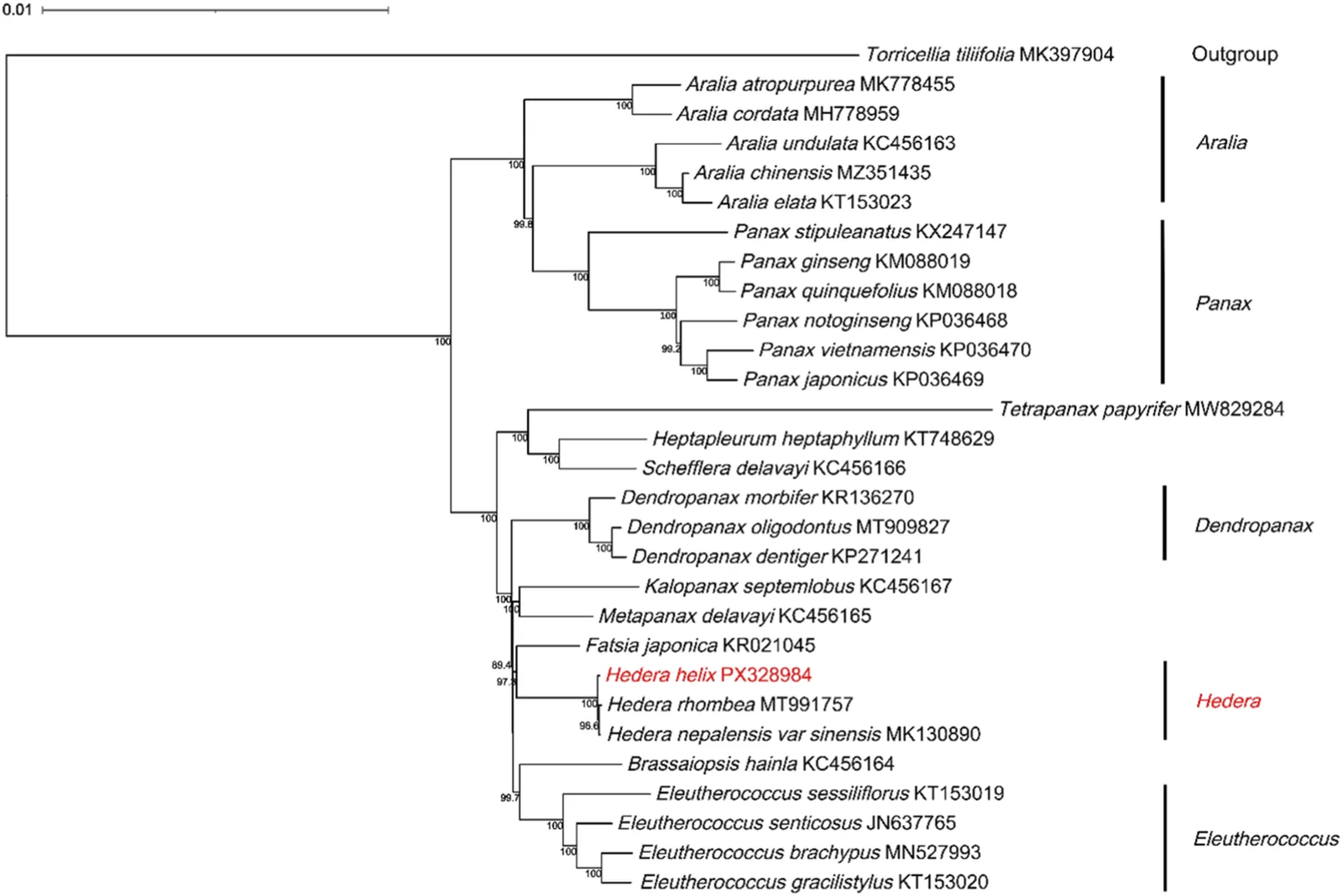
Maximum-likelihood phylogenetic tree inferred from 28 representative Araliaceae plastomes plus *Torricellia tiliifolia* as the outgroup (29 taxa in total). SH-aLRT support values, calculated with 1,000 replicates in IQ-TREE, are shown at the nodes; values ≥80% were interpreted as well supported. Species names with GenBank accession numbers are provided at the tips. Detailed information on the taxa, families, GenBank accession numbers, and usage is provided in Supplementary Table S2.

To clarify the phylogenetic placement of *H. helix*, a maximum-likelihood (ML) tree was reconstructed using 28 representative Araliaceae plastomes, with *Torricellia tiliifolia* as the outgroup (29 taxa in total; Figure 8). The resulting topology showed clear genus-level structure, with major sampled lineages recovered as clades corresponding to *Aralia* (five species), *Panax* (six species), *Dendropanax* (three species), *Hedera* (three species), and *Eleutherococcus* (four species), together with additional representatives of *Tetrapanax*, *Heptapleurum/Schefflera*, *Kalopanax*, *Metapanax*, *Fatsia*, and *Brassaiopsis*. SH-aLRT support values were generally high across the tree (89.4–100.0), indicating strong support for most sampled relationships. Within *Hedera*, *H. helix* formed a clade with *H. rhombea* and *H. nepalensis* var. *sinensis*; *H. rhombea* and *H. nepalensis* var. *sinensis* were resolved as sister taxa (SH-aLRT = 96.6), and *H. helix* was recovered as sister to that pair (SH-aLRT = 100.0). The comparatively short internal branches within the *Hedera* clade, relative to deeper splits among Araliaceae genera, were consistent with limited plastome divergence among the three *Hedera* taxa included in this dataset.

## 4 Discussion

*Hedera helix* is a widely cultivated woody species in Araliaceae and is also used in herbal medicine. Ivy leaf preparations are recognized in Europe for use as an expectorant in productive cough. Plastome-scale datasets are now common in Araliaceae and have improved phylogenetic resolution across major lineages (Kang et al., 2023; Nguyen et al., 2020). A genome sequence note for *H. helix* has been published and provides an important genomic reference (Christenhusz et al., 2023). In that study, the plastid genome was included as an associated organellar assembly, but detailed chloroplast genome annotation and comparative plastome analysis were not the main focus. Therefore, the present study adds a curated chloroplast genome annotation of *H. helix* and a focused comparative framework, including analyses of genome structure, repeats, IR boundaries, Ka/Ks patterns, whole-plastome similarity, structural conservation, nucleotide diversity hotspots, and phylogenetic placement. The plastome characteristics of *H. helix* were also compared with other Araliaceae plastomes and published *Hedera* plastome resources, such as *H. nepalensis* var. *sinensis* (Wu et al., 2019).

The chloroplast genome of *H. helix* showed the typical quadripartite structure of most angiosperm plastomes, including a large single-copy region, a small single-copy region, and two inverted repeats (Daniell et al., 2016; Howe et al., 2003; Wicke et al., 2011). Its overall gene content and gene order were also consistent with the conserved structure reported in many angiosperms, supporting comparative analyses with related Araliaceae plastomes (Daniell et al., 2016; Wicke et al., 2011). Small differences in plastome length among close relatives often reflect expansions or contractions near IR/SC boundaries rather than major rearrangements (Howe et al., 2003; Wicke et al., 2011). These boundary shifts can involve partial duplication of genes at the junctions, such as *ycf1* or *rps19* in many angiosperms, and may generate lineage-specific signatures useful for plastome comparison (Shaw et al., 2007; Wicke et al., 2011). In the present study, a focused comparison of the three available *Hedera* plastomes also showed overall conservation in genome size, GC content, gene content, and IR length, with only minor differences in SSR abundance and localized variable regions (Supplementary Table S5). The mVISTA and Mauve analyses further supported this pattern, indicating that plastome organization is highly conserved among the sampled Araliaceae plastomes, with no major rearrangements detected among the six compared plastomes.

Repeat sequences and chloroplast simple sequence repeats (cpSSRs) are common in plastomes and can provide practical markers for population studies, lineage tracing, and product authentication (Provan et al., 2001; Wheeler et al., 2014). Because cpSSRs are usually nonrecombining and often show uniparental inheritance, they can simplify interpretation in some applications but also limit inference to the organellar lineage (Birky, 1995; Corriveau and Coleman, 1988). In Araliaceae, plastome-derived markers have been used to distinguish closely related taxa and to support authentication frameworks in medicinal genera such as *Panax* (Nguyen et al., 2020). For herbal materials, DNA-based identification is especially useful when diagnostic morphology is lost during processing, and chloroplast markers remain widely used because of their high copy number and stable amplification (de Boer et al., 2015; Raclariu et al., 2018; Techen et al., 2014). Thus, the SSR catalog generated here provides candidate loci for future population sampling of *H. helix* and for routine identification of *Hedera* materials, including medicinal products.

Plastomes contain regions that evolve faster than others, and these hotspots can improve discrimination among closely related species and aid phylogenetic inference at shallow taxonomic levels (Dong et al., 2012; Shaw et al., 2007). The *ycf1* region is among the most variable plastid loci in land plants and has been shown to perform well as a barcode in broad tests (Dong et al., 2015). The core plant barcode *rbcL* + *matK* provides a useful baseline but can have limited resolution in recently diverged groups (CBOL Plant Working Group, 2009). Therefore, hotspot screening in a family-specific context is valuable, especially in Araliaceae, where standard loci can show low divergence among close relatives (Kang et al., 2023; Nguyen et al., 2020). In this study, the highly variable plastome regions detected in *H. helix*, together with the identified SSR loci, provide candidate marker resources for future work on *Hedera* systematics, ivy leaf authentication, and population-level studies. These candidate markers may be useful for assessing maternal-lineage diversity, geographic differentiation, and source tracing among cultivated, naturalized, and wild *H. helix* populations (Wheeler et al., 2014; Feng et al., 2023). However, their diagnostic value and polymorphism should be validated using multiple individuals and populations before routine use in authentication or population genetic studies.

Among the five gene-comparison combinations with Ka/Ks values exceeding 1, *matK* showed the highest value in the comparison between *H. helix* and *Kalopanax septemlobus*. The remaining elevated ratios involved *rpoA* and *atpF* in the comparison with *Dendropanax dentigerus*, *ycf2* in the comparison with *Eleutherococcus brachypus*, and *ycf1* in the comparison with *Metapanax delavayi*. These genes are associated with different plastid functions, including intron splicing (*matK*), plastid transcription (*rpoA*), photosynthetic energy conversion (*atpF*), and large plastid open reading frames such as *ycf1* and *ycf2*. The elevated values for *matK* and *atpF* are noteworthy for two reasons. First, they expand the candidate set of plastid loci showing unusual substitution patterns in the present pairwise comparisons. Second, they indicate that isolated Ka/Ks > 1 estimates should be interpreted cautiously rather than treated as direct evidence of positive selection. Elevated or positive-selection signals involving these loci have also been reported in other plastome studies: *atpF* showed Ka/Ks ratios > 1 among ten *Panax*-related Araliaceae chloroplast genomes (Kim et al., 2017), and *matK* and *atpF* were detected in positive-selection analyses of *Hydrocotyle* plastomes (Wen et al., 2024). In the present study, however, these elevated Ka/Ks ratios were detected only in isolated pairwise contrasts and were not supported by a formal likelihood-based test for positive selection. In addition, because *atpF* is an intron-containing chloroplast gene, annotation errors at exon-intron boundaries or imperfect codon alignment may inflate Ka/Ks estimates (Deshpande et al., 1995; Schneider et al., 2009; Jordan and Goldman, 2012). More broadly, Ka/Ks-based inference should be interpreted cautiously because it can be affected by model assumptions, low sequence divergence, low Ks values, and limited numbers of substitutions (Yang and Bielawski, 2000; Stoletzki and Eyre-Walker, 2011). Therefore, these genes should be regarded as candidate loci with unusual substitution patterns rather than statistically supported positively selected genes. Further analyses based on carefully curated exon boundaries, codon alignments, broader taxon sampling, and likelihood-based branch-site models are needed before any inference of positive selection can be made (Yang and Nielsen, 2002).

The placement of *H. helix* in the present plastome tree should be interpreted as a chloroplast-genome signal rather than a complete species-tree hypothesis. In our analysis, *H. helix* was recovered within the *Hedera* clade and was closely related to the clade formed by *H. rhombea* and *H. nepalensis* var. *sinensis*. This result supports the placement of *H. helix* within *Hedera*, but the exact relationships among these species should be interpreted cautiously. Previous studies of *Hedera* reported incongruence between chloroplast DNA and nuclear ITS phylogenies, suggesting that plastid relationships may not always match nuclear relationships in this genus (Ackerfield and Wen, 2003). Therefore, the relationship recovered here is best viewed as evidence of plastid affinity among the sampled *Hedera* taxa. Whether the same relationship is supported by the nuclear genome remains unresolved in the present study and should be tested using nuclear markers or genome-scale nuclear data in future work. Because the present dataset was designed as representative rather than exhaustive sampling of Araliaceae plastomes, broader taxon sampling may also help refine some deeper intergeneric relationships, although the placement of *H. helix* within *Hedera* is unlikely to be materially altered at the present sampling scale.

Overall, the complete plastome of *H. helix* generated here adds a curated genomic resource for *Hedera* and supports comparative analyses across Araliaceae (Kang et al., 2023; Nguyen et al., 2020). The hotspot loci and cpSSR candidates provide marker options for taxonomy, quality control, and population studies of ivy and its relatives (de Boer et al., 2015; Provan et al., 2001; Techen et al., 2014; Wheeler et al., 2014). Future work should expand sampling across *Hedera* species and populations and integrate nuclear genomic data to test for hybridization and to resolve cytonuclear discordance where it occurs.

## 5 Conclusion

We assembled, annotated, and comparatively analyzed the complete chloroplast genome of *Hedera helix*. Coverage validation supported the reliability of the plastome assembly, and comparative analyses revealed conserved genome organization, informative repeat features, divergence hotspots, and a plastome-based phylogenetic placement of *H. helix* within *Hedera*. This study provides a curated plastome resource for *Hedera*. The identified cpSSR loci and divergence hotspots represent candidate markers for future authentication and population-level studies, while the plastome phylogeny provides a basis for further testing of *Hedera* relationships with broader sampling and nuclear genomic data.

## Data Availability

The datasets presented in this study can be found in online repositories. The names of the repository/repositories and accession number(s) can be found below: https://www.ncbi.nlm.nih.gov/genbank/, PX328984 https://www.ncbi.nlm.nih.gov/, PRJNA1303833 https://www.ncbi.nlm.nih.gov/, SRR34948207 https://www.ncbi.nlm.nih.gov/, SAMN50537044.
