# Supplementary material for: The complete chloroplast genome of Hedera helix (Araliaceae): comparative plastome analyses reveal divergence hotspots, selective constraints, and phylogenetic placement
Source: Front Genet. 2026 Aug 6;17:1855347. doi: 10.3389/fgene.2026.1855347 (PMC13489592; doi:10.3389/fgene.2026.1855347)
Supplement: Supplementary file 1 [file DataSheet1.zip › Supplementary files/Figure S1.docx]

**Figure S1.** Plastome-wide sequencing depth profile of the assembled chloroplast genome of Hedera helix. Clean reads were mapped back to the final plastome assembly, and read depth was calculated across the complete chloroplast genome. Dashed vertical lines indicate the four junctions between the LSC, SSC, and IR regions: JLB, JSB, JSA, and JLA.
