# Supplementary material for: The complete chloroplast genome of Hedera helix (Araliaceae): comparative plastome analyses reveal divergence hotspots, selective constraints, and phylogenetic placement
Source: Front Genet. 2026 Aug 6;17:1855347. doi: 10.3389/fgene.2026.1855347 (PMC13489592; doi:10.3389/fgene.2026.1855347)
Supplement: Supplementary file 1 [file DataSheet1.zip › Supplementary files/Figure S2.docx]

**Figure S2.** Bandage visualization of the selected GetOrganelle assembly graph for the Hedera helix chloroplast genome. The graph was used to inspect the circular assembly structure of the plastome.
