# Supplementary material for: The complete chloroplast genome of Hedera helix (Araliaceae): comparative plastome analyses reveal divergence hotspots, selective constraints, and phylogenetic placement
Source: Front Genet. 2026 Aug 6;17:1855347. doi: 10.3389/fgene.2026.1855347 (PMC13489592; doi:10.3389/fgene.2026.1855347)
Supplement: Supplementary file 1 [file DataSheet1.zip › Supplementary files/Figure S3.docx]

**Figure S3.** Read-depth profiles around the four IR/SC junctions of the Hedera helix chloroplast genome. Read depth was plotted across a 2-kb window centered on each annotated junction. The dashed vertical line indicates the junction position. JLB, LSC/IRb junction; JSB, IRb/SSC junction; JSA, SSC/IRa junction; JLA, IRa/LSC junction.
