# Supplementary material for: The complete chloroplast genome of Hedera helix (Araliaceae): comparative plastome analyses reveal divergence hotspots, selective constraints, and phylogenetic placement
Source: Front Genet. 2026 Aug 6;17:1855347. doi: 10.3389/fgene.2026.1855347 (PMC13489592; doi:10.3389/fgene.2026.1855347)
Supplement: Supplementary file 1 [file DataSheet1.zip › Supplementary files/Figure S4.docx]

**Figure S4.** Whole-plastome comparison between OX381724 and PX328984. Whole-plastome alignment showed that the two *H. helix* plastomes are highly similar and collinear. After removal of the redundant 5,537-bp terminal overlap from OX381724 and rotation to the same starting position as PX328984, the one-to-one alignment covered 100.0% of PX328984 and showed 99.87% nucleotide identity, with 56 SNPs and 25 indel events (12 insertions and 13 deletions; 144 indel-associated bases in total). The terminal 6,056-bp segment of the linear plastid assembly OX381724 aligns with 100% identity to two IR-related positions in the curated circular chloroplast genome PX328984 (86,627-92,682 bp and 150,633-156,688 bp), indicating that the additional length in OX381724 is attributable to a redundant IR-derived terminal segment rather than to a true plastome expansion.
