# Supplementary material for: The complete chloroplast genome of Hedera helix (Araliaceae): comparative plastome analyses reveal divergence hotspots, selective constraints, and phylogenetic placement
Source: Front Genet. 2026 Aug 6;17:1855347. doi: 10.3389/fgene.2026.1855347 (PMC13489592; doi:10.3389/fgene.2026.1855347)
Supplement: Supplementary file 1 [file DataSheet1.zip › Supplementary files/Figure S5.docx]

**Figure S5.** Mauve-based collinearity comparison of complete chloroplast genomes from Hedera helix and selected related Araliaceae species. The plastomes were normalized to the psbA starting position before alignment. No major rearrangements or inversions were detected among the compared plastomes.
