# Supplementary material for: The complete chloroplast genome of Hedera helix (Araliaceae): comparative plastome analyses reveal divergence hotspots, selective constraints, and phylogenetic placement
Source: Front Genet. 2026 Aug 6;17:1855347. doi: 10.3389/fgene.2026.1855347 (PMC13489592; doi:10.3389/fgene.2026.1855347)
Supplement: Supplementary file 1 [file DataSheet1.zip › Supplementary files/Table S1.docx]

Table S1. Sliding-window plastome hotspots in six Araliaceae plastomes

| Window | Alignment window | *H. helix* start | *H. helix* end | Pi | Net sites | Associated region | Region type |
| --- | --- | --- | --- | --- | --- | --- | --- |
| W010 | 1801-2400 | 1638 | 2211 | 0.01167542 | 571 | matK/trnK-UUU | genic |
| W011 | 2001-2600 | 1821 | 2411 | 0.01018676 | 589 | matK/trnK-UUU | genic |
| W022 | 4201-4800 | 4009 | 4588 | 0.01089631 | 569 | trnK-UUU | genic |
| W023 | 4401-5000 | 4209 | 4787 | 0.01440781 | 546 | trnK-UUU | genic |
| W024 | 4601-5200 | 4393 | 4986 | 0.01448763 | 566 | trnK-UUU-rps16 | intergenic |
| W025 | 4801-5400 | 4589 | 5147 | 0.01245136 | 514 | trnK-UUU-rps16 | intergenic |
| W026 | 5001-5600 | 4788 | 5347 | 0.01318267 | 531 | rps16 | genic |
| W027 | 5201-5800 | 4987 | 5545 | 0.01188243 | 533 | rps16 | genic |
| W028 | 5401-6000 | 5148 | 5742 | 0.01078932 | 587 | rps16 | genic |
| W034 | 6601-7200 | 6322 | 6908 | 0.01362007 | 558 | rps16 | genic |
| W035 | 6801-7400 | 6518 | 7101 | 0.01570743 | 556 | rps16-trnQ-UUG | intergenic |
| W036 | 7001-7600 | 6710 | 7300 | 0.01474820 | 556 | rps16-trnQ-UUG | intergenic |
| W037 | 7201-7800 | 6909 | 7457 | 0.02007576 | 528 | rps16-trnQ-UUG | intergenic |
| W038 | 7401-8000 | 7102 | 7653 | 0.01480100 | 536 | rps16-trnQ-UUG | intergenic |
| W039 | 7601-8200 | 7301 | 7846 | 0.01880342 | 546 | rps16-trnQ-UUG | intergenic |
| W040 | 7801-8400 | 7458 | 8046 | 0.01077098 | 588 | rps16-trnQ-UUG | intergenic |
| W047 | 9201-9800 | 8835 | 9428 | 0.01069820 | 592 | psbI/trnS-GCU | genic |
| W051 | 10001-10600 | 9617 | 10171 | 0.01139706 | 544 | trnS-GCU-trnG-UCC | intergenic |
| W052 | 10201-10800 | 9802 | 10371 | 0.01244131 | 568 | trnG-UCC | genic |
| W053 | 10401-11000 | 9972 | 10561 | 0.01452258 | 583 | trnG-UCC | genic |
| W054 | 10601-11200 | 10172 | 10761 | 0.01246427 | 583 | trnG-UCC | genic |
| W055 | 10801-11400 | 10372 | 10961 | 0.01226852 | 576 | trnG-UCC | genic |
| W067 | 13201-13800 | 12725 | 13322 | 0.01204013 | 598 | atpF/atpA | genic |
| W068 | 13401-14000 | 12923 | 13522 | 0.01311111 | 600 | atpF | genic |
| W073 | 14401-15000 | 13921 | 14499 | 0.01005848 | 570 | atpF | genic |
| W074 | 14601-15200 | 14121 | 14699 | 0.01076023 | 570 | atpH/atpF | genic |
| W079 | 15601-16200 | 15100 | 15683 | 0.01083628 | 566 | atpH-atpI | intergenic |
| W080 | 15801-16400 | 15296 | 15883 | 0.01461988 | 570 | atpH-atpI | intergenic |
| W081 | 16001-16600 | 15492 | 16082 | 0.01176471 | 578 | atpI | genic |
| W146 | 29001-29600 | 28448 | 29047 | 0.01204819 | 581 | rpoB-trnC-GCA | intergenic |
| W147 | 29201-29800 | 28648 | 29247 | 0.01660900 | 578 | rpoB-trnC-GCA | intergenic |
| W148 | 29401-30000 | 28848 | 29447 | 0.01854380 | 586 | rpoB-trnC-GCA | intergenic |
| W149 | 29601-30200 | 29048 | 29647 | 0.01308090 | 581 | trnC-GCA | genic |
| W151 | 30001-30600 | 29448 | 30005 | 0.01074074 | 540 | trnC-GCA | genic |
| W152 | 30201-30800 | 29648 | 30205 | 0.01275046 | 549 | trnC-GCA-petN | intergenic |
| W153 | 30401-31000 | 29843 | 30405 | 0.01107100 | 554 | petN | genic |
| W156 | 31001-31600 | 30406 | 30984 | 0.01370178 | 579 | petN-psbM | intergenic |
| W158 | 31401-32000 | 30801 | 31348 | 0.01088180 | 533 | psbM | genic |
| W163 | 32401-33000 | 31730 | 32326 | 0.01015982 | 584 | psbM-trnD-GUC | intergenic |
| W168 | 33401-34000 | 32726 | 33261 | 0.01835749 | 483 | trnE-UUC/trnY-GUA | genic |
| W169 | 33601-34200 | 32926 | 33460 | 0.02217544 | 475 | trnE-UUC-trnT-GGU | intergenic |
| W170 | 33801-34400 | 33095 | 33660 | 0.01463104 | 524 | trnE-UUC-trnT-GGU | intergenic |
| W177 | 35201-35800 | 34436 | 35033 | 0.01122449 | 588 | trnT-GGU-psbD | intergenic |
| W178 | 35401-36000 | 34634 | 35232 | 0.01098039 | 595 | psbD | genic |
| W226 | 45001-45600 | 44189 | 44785 | 0.01009070 | 588 | psaA-ycf3 | intergenic |
| W240 | 47801-48400 | 46954 | 47548 | 0.01130137 | 584 | ycf3-trnS-GGA | intergenic |
| W241 | 48001-48600 | 47150 | 47742 | 0.01289954 | 584 | trnS-GGA | genic |
| W242 | 48201-48800 | 47350 | 47942 | 0.01050228 | 584 | trnS-GGA | genic |
| W249 | 49601-50200 | 48743 | 49325 | 0.01203704 | 504 | trnT-UGU | genic |
| W250 | 49801-50400 | 48943 | 49525 | 0.01232493 | 476 | trnT-UGU | genic |
| W251 | 50001-50600 | 49143 | 49713 | 0.01034483 | 464 | trnT-UGU-trnL-UAA | intergenic |
| W271 | 54001-54600 | 52963 | 53536 | 0.01138498 | 568 | ndhC-trnV-UAC | intergenic |
| W272 | 54201-54800 | 53163 | 53736 | 0.01149826 | 574 | ndhC-trnV-UAC | intergenic |
| W273 | 54401-55000 | 53341 | 53936 | 0.01073446 | 590 | trnV-UAC | genic |
| W278 | 55401-56000 | 54335 | 54933 | 0.01237458 | 598 | trnM-CAU/trnV-UAC | genic |
| W279 | 55601-56200 | 54535 | 55133 | 0.01518705 | 597 | atpE/trnM-CAU/trnV-UAC | genic |
| W280 | 55801-56400 | 54735 | 55333 | 0.01563372 | 597 | atpE/trnM-CAU | genic |
| W291 | 58001-58600 | 56934 | 57504 | 0.01191740 | 565 | atpB | genic |
| W292 | 58201-58800 | 57134 | 57697 | 0.01033033 | 555 | atpB-rbcL | intergenic |
| W302 | 60201-60800 | 59098 | 59686 | 0.01312057 | 564 | rbcL | genic |
| W334 | 66601-67200 | 65425 | 66019 | 0.01165266 | 595 | petA-psbJ | intergenic |
| W335 | 66801-67400 | 65625 | 66214 | 0.01209040 | 590 | petA-psbJ | intergenic |
| W336 | 67001-67600 | 65825 | 66414 | 0.01059829 | 585 | psbJ | genic |
| W343 | 68401-69000 | 67215 | 67813 | 0.01445313 | 512 | psbE-petL | intergenic |
| W344 | 68601-69200 | 67414 | 68013 | 0.02786325 | 390 | psbE-petL | intergenic |
| W345 | 68801-69400 | 67614 | 68213 | 0.02034188 | 390 | psbE-petL | intergenic |
| W346 | 69001-69600 | 67814 | 68410 | 0.01390681 | 465 | petL | genic |
| W355 | 70801-71400 | 69611 | 70204 | 0.01596402 | 593 | rpl33 | genic |
| W356 | 71001-71600 | 69806 | 70393 | 0.01484018 | 584 | rpl33 | genic |
| W357 | 71201-71800 | 70006 | 70593 | 0.01426941 | 584 | rpl33/rps18 | genic |
| W393 | 78401-79000 | 77129 | 77720 | 0.01137931 | 580 | psbH/petB | genic |
| W408 | 81401-82000 | 80109 | 80698 | 0.01050228 | 584 | rpoA/petD | genic |
| W412 | 82201-82800 | 80899 | 81497 | 0.01057318 | 599 | rpoA/rps11 | genic |
| W430 | 85801-86400 | 84486 | 85085 | 0.01228669 | 586 | rpl16 | genic |
| W431 | 86001-86600 | 84686 | 85285 | 0.01549815 | 542 | rps3/rpl16 | genic |
| W432 | 86201-86800 | 84886 | 85485 | 0.01266913 | 542 | rps3/rpl16 | genic |
| W582 | 116201-116800 | 114831 | 115424 | 0.01065292 | 582 | ndhF-rpl32 | intergenic |
| W583 | 116401-117000 | 115031 | 115602 | 0.01214286 | 560 | ndhF-rpl32 | intergenic |
| W584 | 116601-117200 | 115231 | 115778 | 0.01180049 | 548 | ndhF-rpl32 | intergenic |
| W585 | 116801-117400 | 115425 | 115977 | 0.01198777 | 545 | ndhF-rpl32 | intergenic |
| W589 | 117601-118200 | 116178 | 116763 | 0.01415205 | 570 | rpl32-trnL-UAG | intergenic |
| W590 | 117801-118400 | 116378 | 116962 | 0.01294050 | 577 | rpl32-trnL-UAG | intergenic |
| W591 | 118001-118600 | 116565 | 117162 | 0.01027359 | 597 | ccsA/trnL-UAG | genic |
| W615 | 122801-123400 | 121363 | 121954 | 0.01291811 | 578 | ndhG/ndhI | genic |
| W616 | 123001-123600 | 121563 | 122154 | 0.01234141 | 578 | ndhI | genic |
| W642 | 128201-128800 | 126745 | 127344 | 0.01511111 | 600 | ycf1 | genic |
| W643 | 128401-129000 | 126945 | 127529 | 0.01595442 | 585 | ycf1 | genic |
| W644 | 128601-129200 | 127145 | 127723 | 0.02993667 | 579 | ycf1 | genic |
| W645 | 128801-129400 | 127345 | 127923 | 0.02602188 | 579 | ycf1 | genic |
| W646 | 129001-129600 | 127530 | 128120 | 0.02549351 | 591 | ycf1 | genic |
| W647 | 129201-129800 | 127724 | 128320 | 0.01563372 | 597 | ycf1 | genic |
| W648 | 129401-130000 | 127924 | 128520 | 0.01579244 | 591 | ycf1 | genic |
| W649 | 129601-130200 | 128121 | 128714 | 0.01247166 | 588 | ycf1 | genic |
| W651 | 130001-130600 | 128521 | 129114 | 0.01258503 | 588 | ycf1 | genic |
| W652 | 130201-130800 | 128715 | 129314 | 0.01324355 | 594 | ycf1 | genic |
| W653 | 130401-131000 | 128915 | 129514 | 0.01833333 | 600 | ycf1 | genic |
| W654 | 130601-131200 | 129115 | 129714 | 0.01633333 | 600 | ycf1 | genic |
| W655 | 130801-131400 | 129315 | 129913 | 0.02025598 | 599 | ycf1 | genic |
| W656 | 131001-131600 | 129515 | 130105 | 0.01928934 | 591 | ycf1 | genic |
| W657 | 131201-131800 | 129715 | 130305 | 0.02143260 | 591 | ycf1 | genic |
| W658 | 131401-132000 | 129914 | 130505 | 0.02117117 | 592 | ycf1 | genic |
| W659 | 131601-132200 | 130106 | 130705 | 0.02177778 | 600 | ycf1 | genic |
| W660 | 131801-132400 | 130306 | 130905 | 0.01777778 | 600 | ycf1 | genic |
| W661 | 132001-132600 | 130506 | 131105 | 0.01133333 | 600 | ycf1 | genic |

Pi was calculated from the MAFFT-aligned complete plastomes using a 600 bp window and 200 bp step size. Windows with Pi > 0.01 are listed below. Coordinates are mapped to the *H. helix* plastome (PX328984).

Merged hotspot regions

| Hotspot ID | Alignment region | *H. helix* start | *H. helix* end | Max Pi | Windows | Associated region |
| --- | --- | --- | --- | --- | --- | --- |
| HS01 | 1801-2600 | 1638 | 2411 | 0.01167542 | 2 | matK/trnK-UUU |
| HS02 | 4201-6000 | 4009 | 5742 | 0.01448763 | 7 | rps16/trnK-UUU |
| HS03 | 6601-8400 | 6322 | 8046 | 0.02007576 | 7 | rps16 |
| HS04 | 9201-9800 | 8835 | 9428 | 0.01069820 | 1 | psbI/trnS-GCU |
| HS05 | 10001-11400 | 9617 | 10961 | 0.01452258 | 5 | trnG-UCC |
| HS06 | 13201-14000 | 12725 | 13522 | 0.01311111 | 2 | atpF/atpA |
| HS07 | 14401-15200 | 13921 | 14699 | 0.01076023 | 2 | atpF/atpH |
| HS08 | 15601-16600 | 15100 | 16082 | 0.01461988 | 3 | atpI |
| HS09 | 29001-32000 | 28448 | 31348 | 0.01854380 | 9 | psbM/petN/trnC-GCA |
| HS10 | 32401-33000 | 31730 | 32326 | 0.01015982 | 1 | psbM-trnD-GUC |
| HS11 | 33401-34400 | 32726 | 33660 | 0.02217544 | 3 | trnE-UUC/trnY-GUA |
| HS12 | 35201-36000 | 34436 | 35232 | 0.01122449 | 2 | psbD |
| HS13 | 45001-45600 | 44189 | 44785 | 0.01009070 | 1 | psaA-ycf3 |
| HS14 | 47801-48800 | 46954 | 47942 | 0.01289954 | 3 | trnS-GGA |
| HS15 | 49601-50600 | 48743 | 49713 | 0.01232493 | 3 | trnT-UGU |
| HS16 | 54001-55000 | 52963 | 53936 | 0.01149826 | 3 | trnV-UAC |
| HS17 | 55401-56400 | 54335 | 55333 | 0.01563372 | 3 | atpE/trnM-CAU/trnV-UAC |
| HS18 | 58001-58800 | 56934 | 57697 | 0.01191740 | 2 | atpB |
| HS19 | 60201-60800 | 59098 | 59686 | 0.01312057 | 1 | rbcL |
| HS20 | 66601-67600 | 65425 | 66414 | 0.01209040 | 3 | psbJ |
| HS21 | 68401-69600 | 67215 | 68410 | 0.02786325 | 4 | petL |
| HS22 | 70801-71800 | 69611 | 70593 | 0.01596402 | 3 | rpl33/rps18 |
| HS23 | 78401-79000 | 77129 | 77720 | 0.01137931 | 1 | psbH/petB |
| HS24 | 81401-82000 | 80109 | 80698 | 0.01050228 | 1 | rpoA/petD |
| HS25 | 82201-82800 | 80899 | 81497 | 0.01057318 | 1 | rpoA/rps11 |
| HS26 | 85801-86800 | 84486 | 85485 | 0.01549815 | 3 | rps3/rpl16 |
| HS27 | 116201-117400 | 114831 | 115977 | 0.01214286 | 4 | ndhF-rpl32 |
| HS28 | 117601-118600 | 116178 | 117162 | 0.01415205 | 3 | ccsA/trnL-UAG |
| HS29 | 122801-123600 | 121363 | 122154 | 0.01291811 | 2 | ndhI/ndhG |
| HS30 | 128201-132600 | 126745 | 131105 | 0.02993667 | 19 | ycf1 |
