# Supplementary material for: The complete chloroplast genome of Hedera helix (Araliaceae): comparative plastome analyses reveal divergence hotspots, selective constraints, and phylogenetic placement
Source: Front Genet. 2026 Aug 6;17:1855347. doi: 10.3389/fgene.2026.1855347 (PMC13489592; doi:10.3389/fgene.2026.1855347)
Supplement: Supplementary file 1 [file DataSheet1.zip › Supplementary files/Table S2.docx]

Table S2. Taxa and GenBank accession numbers of chloroplast genomes used for phylogenetic analysis.

| No. | Taxon | Family | GenBank accession No. | Use |
| --- | --- | --- | --- | --- |
| 1 | *Torricellia tiliifolia* | Torricelliaceae | MK397904 | Outgroup |
| 2 | *Aralia atropurpurea* | Araliaceae | MK778455 | Ingroup |
| 3 | *Aralia cordata* | Araliaceae | MH778959 | Ingroup |
| 4 | *Aralia undulata* | Araliaceae | KC456163 | Ingroup |
| 5 | *Aralia chinensis* | Araliaceae | MZ351435 | Ingroup |
| 6 | *Aralia elata* | Araliaceae | KT153023 | Ingroup |
| 7 | *Panax stipuleanatus* | Araliaceae | KX247147 | Ingroup |
| 8 | *Panax ginseng* | Araliaceae | KM088019 | Ingroup |
| 9 | *Panax quinquefolius* | Araliaceae | KM088018 | Ingroup |
| 10 | *Panax notoginseng* | Araliaceae | KP036468 | Ingroup |
| 11 | *Panax vietnamensis* | Araliaceae | KP036470 | Ingroup |
| 12 | *Panax japonicus* | Araliaceae | KP036469 | Ingroup |
| 13 | *Tetrapanax papyrifer* | Araliaceae | MW829284 | Ingroup |
| 14 | *Heptapleurum heptaphyllum* | Araliaceae | KT748629 | Ingroup |
| 15 | *Schefflera delavayi* | Araliaceae | KC456166 | Ingroup |
| 16 | *Dendropanax morbifer* | Araliaceae | KR136270 | Ingroup |
| 17 | *Dendropanax oligodontus* | Araliaceae | MT909827 | Ingroup |
| 18 | *Dendropanax dentigerus* | Araliaceae | KP271241 | Ingroup |
| 19 | *Kalopanax septemlobus* | Araliaceae | KC456167 | Ingroup |
| 20 | *Metapanax delavayi* | Araliaceae | KC456165 | Ingroup |
| 21 | *Fatsia japonica* | Araliaceae | KR021045 | Ingroup |
| 22 | *Hedera helix* | Araliaceae | PX328984 | Ingroup |
| 23 | *Hedera rhombea* | Araliaceae | MT991757 | Ingroup |
| 24 | *Hedera nepalensis* var. *sinensis* | Araliaceae | MK130890 | Ingroup |
| 25 | *Brassaiopsis hainla* | Araliaceae | KC456164 | Ingroup |
| 26 | *Eleutherococcus sessiliflorus* | Araliaceae | KT153019 | Ingroup |
| 27 | *Eleutherococcus senticosus* | Araliaceae | JN637765 | Ingroup |
| 28 | *Eleutherococcus brachypus* | Araliaceae | MN527993 | Ingroup |
| 29 | *Eleutherococcus gracilistylus* | Araliaceae | KT153020 | Ingroup |
