# Supplementary material for: The complete chloroplast genome of Hedera helix (Araliaceae): comparative plastome analyses reveal divergence hotspots, selective constraints, and phylogenetic placement
Source: Front Genet. 2026 Aug 6;17:1855347. doi: 10.3389/fgene.2026.1855347 (PMC13489592; doi:10.3389/fgene.2026.1855347)
Supplement: Supplementary file 1 [file DataSheet1.zip › Supplementary files/Table S3.docx]

Table S3. Comparison between the previously reported *Hedera helix* plastid genome and the chloroplast genome generated in this study.

| Item | Christenhusz et al. (2023) | Present study |
| --- | --- | --- |
| Study focus | Chromosome-level genome assembly of *H. helix* | Focused chloroplast genome characterization and comparative plastome analysis |
| Plastid/chloroplast accession | OX381724 | PX328984 |
| Genome length | 162,225 bp | 156,688 bp |
| Genome form | Linear plastid genome record | Circular chloroplast genome |
| Gene annotation | Not the focus of that study | 132 genes, including 87 PCGs, 37 tRNAs, and 8 rRNAs |
| Comparative analyses | Not systematically analyzed | SSRs, long repeats, IR/SC boundaries, Pi hotspots, Ka/Ks, synteny, and phylogeny |
| Main added value | Provides a genome assembly resource | Provides a curated chloroplast genome and comparative plastome framework |
