# Supplementary material for: The complete chloroplast genome of Hedera helix (Araliaceae): comparative plastome analyses reveal divergence hotspots, selective constraints, and phylogenetic placement
Source: Front Genet. 2026 Aug 6;17:1855347. doi: 10.3389/fgene.2026.1855347 (PMC13489592; doi:10.3389/fgene.2026.1855347)
Supplement: Supplementary file 1 [file DataSheet1.zip › Supplementary files/Table S4.docx]

Table S4. Simple sequence repeats identified in the chloroplast genome of *Hedera helix*.

| No. | SSR type | Motif | Repeat number | Start position | End position | Length (bp) | Plastome region | Location / gene |
| --- | --- | --- | --- | --- | --- | --- | --- | --- |
| 1 | Tetranucleotide | AATA | 3 | 4357 | 4368 | 12 | LSC | IGS(trnK-UUU, rps16) |
| 2 | Mononucleotide | A | 11 | 4800 | 4810 | 11 | LSC | IGS(trnK-UUU, rps16) |
| 3 | Mononucleotide | T | 10 | 6894 | 6903 | 10 | LSC | IGS(rps16, trnQ-UUG) |
| 4 | Mononucleotide | T | 10 | 9106 | 9115 | 10 | LSC | IGS(psbK, psbI) |
| 5 | Mononucleotide | T | 10 | 13572 | 13581 | 10 | LSC | atpF(intron) |
| 6 | Mononucleotide | A | 13 | 14194 | 14206 | 13 | LSC | IGS(atpF, atpH) |
| 7 | Mononucleotide | A | 11 | 17639 | 17649 | 11 | LSC | IGS(rps2, rpoC2) |
| 8 | Mononucleotide | T | 11 | 19858 | 19868 | 11 | LSC | rpoC2 |
| 9 | Dinucleotide | AT | 6 | 22260 | 22271 | 12 | LSC | IGS(rpoC2, rpoC1) |
| 10 | Mononucleotide | C | 10 | 23915 | 23924 | 10 | LSC | IGS(rpoC2, rpoC1) |
| 11 | Mononucleotide | A | 10 | 23925 | 23934 | 10 | LSC | IGS(rpoC2, rpoC1) |
| 12 | Mononucleotide | T | 10 | 27572 | 27581 | 10 | LSC | rpoB |
| 13 | Dinucleotide | TA | 6 | 29893 | 29904 | 12 | LSC | IGS(trnC-GCA, petN) |
| 14 | Dinucleotide | TA | 5 | 29928 | 29937 | 10 | LSC | IGS(trnC-GCA, petN) |
| 15 | Tetranucleotide | AAGA | 3 | 31045 | 31056 | 12 | LSC | IGS(petN, psbM) |
| 16 | Tetranucleotide | TCTT | 3 | 31067 | 31078 | 12 | LSC | IGS(petN, psbM) |
| 17 | Tetranucleotide | AATT | 3 | 31211 | 31222 | 12 | LSC | IGS(petN, psbM) |
| 18 | Dinucleotide | TA | 5 | 33620 | 33629 | 10 | LSC | IGS(trnE-UUC, trnT-GGU) |
| 19 | Mononucleotide | C | 10 | 38449 | 38458 | 10 | LSC | IGS(psbZ, trnG-GCC) |
| 20 | Mononucleotide | A | 11 | 46962 | 46972 | 11 | LSC | IGS(ycf3, trnS-GGA) |
| 21 | Mononucleotide | T | 10 | 56884 | 56893 | 10 | LSC | atpB |
| 22 | Trinucleotide | ATA | 4 | 56938 | 56949 | 12 | LSC | atpB |
| 23 | Mononucleotide | A | 10 | 57485 | 57494 | 10 | LSC | IGS(atpB, rbcL) |
| 24 | Dinucleotide | AT | 5 | 59249 | 59258 | 10 | LSC | IGS(rbcL, accD) |
| 25 | Mononucleotide | A | 10 | 61566 | 61575 | 10 | LSC | IGS(accD, psaI) |
| 26 | Mononucleotide | A | 10 | 63326 | 63335 | 10 | LSC | IGS(ycf4, cemA) |
| 27 | Trinucleotide | TTC | 4 | 69972 | 69983 | 12 | LSC | IGS(psaJ, rpl33) |
| 28 | Dinucleotide | TA | 5 | 70293 | 70302 | 10 | LSC | IGS(rpl33, rps18) |
| 29 | Tetranucleotide | TATT | 3 | 70332 | 70343 | 12 | LSC | IGS(rpl33, rps18) |
| 30 | Tetranucleotide | AAAG | 3 | 72676 | 72687 | 12 | LSC | clpP(intron) |
| 31 | Mononucleotide | T | 11 | 72975 | 72985 | 11 | LSC | clpP(intron) |
| 32 | Mononucleotide | A | 13 | 73138 | 73150 | 13 | LSC | clpP(intron) |
| 33 | Mononucleotide | T | 10 | 80582 | 80591 | 10 | LSC | rpoA |
| 34 | Mononucleotide | T | 10 | 82522 | 82531 | 10 | LSC | IGS(infA, rps8) |
| 35 | Mononucleotide | T | 10 | 83633 | 83642 | 10 | LSC | IGS(rpl14, rpl16) |
| 36 | Dinucleotide | TA | 7 | 86365 | 86378 | 14 | LSC | IGS(rpl22, rps19) |
| 37 | Pentanucleotide | ATTAG | 3 | 101296 | 101310 | 15 | IR | IGS(rps7, trnV-GAC) |
| 38 | Mononucleotide | G | 10 | 105964 | 105973 | 10 | IR | trnA-UGC(intron) |
| 39 | Tetranucleotide | AGGT | 3 | 108047 | 108058 | 12 | IR | rrn23 |
| 40 | Dinucleotide | TA | 6 | 111010 | 111021 | 12 | IR | IGS(trnN-GUU, ndhF) |
| 41 | Mononucleotide | A | 10 | 116336 | 116345 | 10 | SSC | IGS(rpl32, trnL-UAG) |
| 42 | Tetranucleotide | ATTA | 3 | 121608 | 121619 | 12 | SSC | IGS(ndhG, ndhI) |
| 43 | Mononucleotide | T | 11 | 129025 | 129035 | 11 | SSC | ycf1 |
| 44 | Dinucleotide | AT | 6 | 132293 | 132304 | 12 | IR | IGS(ycf1, trnN-GUU) |
| 45 | Tetranucleotide | CTAC | 3 | 135255 | 135266 | 12 | IR | rrn23 |
| 46 | Mononucleotide | C | 10 | 137342 | 137351 | 10 | IR | trnA-UGC(intron) |
| 47 | Pentanucleotide | CTAAT | 3 | 142005 | 142019 | 15 | IR | IGS(trnV-GAC, rps7) |

LSC, large single-copy region; SSC, small single-copy region; IR, inverted repeat region; IGS, intergenic spacer.
