# Supplementary material for: The complete chloroplast genome of Hedera helix (Araliaceae): comparative plastome analyses reveal divergence hotspots, selective constraints, and phylogenetic placement
Source: Front Genet. 2026 Aug 6;17:1855347. doi: 10.3389/fgene.2026.1855347 (PMC13489592; doi:10.3389/fgene.2026.1855347)
Supplement: Supplementary file 1 [file DataSheet1.zip › Supplementary files/Table S5.docx]

Table S5. Comparative plastome features of three *Hedera* species

| **Species** | **Accession** | **Genome size (bp)** | **GC (%)** | **LSC (bp)** | **SSC (bp)** | **IR (bp)** | **Gene content** | **SSR abundance (MISA)** | **Relatively variable regions** |
| --- | --- | --- | --- | --- | --- | --- | --- | --- | --- |
| *Hedera helix* | PX328984 | 156,688 | 37.99 | 86,626 | 18,180 | 25,941 | 132 (87 PCGs, 37 tRNAs, 8 rRNAs) | 47 (mono 25; di 9; tri 2; tetra 9; penta 2; hexa 0) | matK/trnK-UUU (1,800-2,799 bp, max Pi=0.00222);  petB/petD (78,761-79,760 bp, max Pi=0.00222);  ycf1 (128,361-129,360 bp, max Pi=0.00222) |
| *Hedera rhombea* | MT991757 | 156,680 | 38.00 | 86,625 | 18,173 | 25,941 | 132 (87 PCGs, 37 tRNAs, 8 rRNAs) | 48 (mono 26; di 9; tri 2; tetra 9; penta 2; hexa 0) |  |
| *Hedera nepalensis* var. sinensis | MK130890 | 156,652 | 38.00 | 86,597 | 18,173 | 25,941 | 132 (87 PCGs, 37 tRNAs, 8 rRNAs) | 49 (mono 27; di 9; tri 2; tetra 9; penta 2; hexa 0) |  |

Gene content counts include duplicated IR copies. SSRs were identified with MISA using thresholds of 10, 5, 4, 3, 3, and 3 repeat units for mono-, di-, tri-, tetra-, penta-, and hexanucleotide motifs, respectively, with a 100 bp maximum distance for compound SSRs. IR length was detected as the longest exact palindromic repeat using Vmatch; the values for *H. helix* and *H. nepalensis* var. *sinensis* match the previous IRscope output. Relatively variable regions were defined from the three-*Hedera* MAFFT alignment using a 600 bp window and 200 bp step; merged windows with max Pi >= 0.002 are listed.
